# B Cells and Programmed Death-Ligand 2 Signaling Are Required for Maximal Interferon-γ Recall Response by Splenic CD4^+^ Memory T Cells of Mice Vaccinated with *Mycobacterium tuberculosis* Ag85B

**DOI:** 10.1371/journal.pone.0137783

**Published:** 2015-09-17

**Authors:** Antonella Riccomi, Carla Palma

**Affiliations:** Department of Infectious, Parasitic and Immune-mediated Diseases, Istituto Superiore di Sanità, Viale Regina Elena, 299, 00161, Rome, Italy; The Catholic University of the Sacred Heart, Rome, ITALY

## Abstract

CD4^+^ T cells producing interferon-γ are crucial for protection against *Mycobacterium tuberculosis* infection and are the cornerstone of tuberculosis vaccination and immunological diagnostic assays. Since emerging evidence indicates that B cells can modulate T cell responses to *M*. *tuberculosis* infection, we investigated the contribution of B cells in regulating interferon-γ recall response by memory Thelper1 cells specific for Ag85B, a leading candidate for tuberculosis sub-unit vaccines. We found that B cells were able to maximize the reactivation of CD4^+^ memory T cells and the interferon-γ response against *ex vivo* antigen recall in spleens of mice vaccinated with Ag85B. B cell-mediated increase of interferon-γ response was particular evident for high interferon-γ producer CD4^+^ memory T cells, likely because those T cells were required for triggering and amplification of B cell activation. A positive-feedback loop of mutual activation between B cells, not necessarily antigen-experienced but with integral phosphatidylinositol-3 kinase (PI3K) pathway and a peculiar interferon-γ-producing CD4^high^T cell subset was established. Programed death-ligand 2 (PD-L2), expressed both on B and the highly activated CD4^high^ T cells, contributed to the increase of interferon-γ recall response through a PD1-independent pathway. In B cell-deficient mice, interferon-γ production and activation of Ag85B-specific CD4^+^ T cells were blunted against *ex vivo* antigen recall but these responses could be restored by adding B cells. On the other hand, B cells appeared to down-regulate interleukin-22 recall response. Our data point out that nature of antigen presenting cells determines quality and size of T cell cytokine recall responses. Thus, antigen presenting cells, including B cells, deserve to be considered for a better prediction of cytokine responses by peripheral memory T cells specific for *M*. *tuberculosis* antigens. We also invite to consider B cells, PD-L2 and PI3K as potential targets for therapeutic modulation of T cell cytokine responses for tuberculosis control.

## Introduction

Tuberculosis (TB) causes 1.8 million deaths annually, and one-third of the world population is latently infected with *Mycobacterium tuberculosis* (Mtb). Coupled with the emergence of multidrug-resistant Mtb strains and the failure of the current bacille Calmette-Guérin (BCG) vaccine to consistently protect against the pulmonary, transmissible form of the disease, this makes TB a worldwide human threat. Thus, the generation of a fully protective vaccine is a top priority in the current list of major medical needs. Many new vaccine formulations have been generated, and some of them are in clinical trial [1] (http://clinicaltrials.gov/ct2/show/NCT00953927?cntry1=AF%3AZA&phase=1&rank=136). Interferon-γ (IFN-γ) signaling Thelper (Th)1 axis is crucial in protection against Mtb infection [2, 3]. Although not a true correlate of protection, the evaluation of IFN-γ response to recall Mtb antigens by peripheral CD4^+^ memory T cells is widely used to test immunogenicity and efficacy of TB vaccines in both mice and humans [4–6]. Moreover, IFN-γ produced by peripheral CD4^+^ effector/memory T cells in response to Mtb recall antigens is commonly used for diagnosis of latent/active Mtb infection [7, 8], to detect clinical progression of TB [9–12] and, more in general, to study the outcome of Mtb infections [13–15]. Ag85B (30kDA), the most abundant extracellular protein of Mtb released during natural infection [16], has a high affinity for T-cell recognition and can induce a protective Th1 immune response [17–19]. For these reasons, Ag85B is a leading candidate for TB sub-unit vaccines [20, 21] (http://www.clinicaltrials.gov/show/NCT01049282). However, high levels of IFN-γ released by Ag85B-specific CD4^+^ T cells have been sometimes associated with a more severe pathology [22, 23] and interference with development of protective immunity during experimental vaccination [17, 18, 24, 25]. In this context, the knowledge of the cellular and molecular mechanisms regulating the IFN-γ recall by Ag85B-specific CD4^+^ memory T cells is essential for a thorough understanding of the immune response evoked by vaccination and/or Mtb infection.

B cells are gaining prominence as modulators of CD4^+^ T cell responses [26]. Recent data from patients and mouse models showing that B cells, beyond antibody (Ab) production, affect antigen presentation, cytokine production, co-stimulation and development of lymphoid tissue architecture, which are directly involved in priming [27, 28] and maintenance of CD4^+^ memory T cells in both infectious and autoimmune diseases [29–31]. Even in the mechanisms of protection from TB, B cells, for long thought to be inconsistent [32], have been re-valued. B-cell-deficient mice show an exacerbated immunopathology associated with elevated pulmonary recruitment of neutrophils during acute phase [33–35] and a delay of inflammatory progression during the chronic phase of the Mtb-infection [36].

Although B cells are required for a right development of Th1 responses induced by BCG vaccination in mice [34, 35], it is still unclear whether these lymphocytes regulate cytokine recall responses by memory CD4^+^ T cells. Here, we address this issue in a mouse model of TB vaccination. Both wild-type (WT) and B cell-deficient C57BL/6 mice were immunized with two different Ag85B-based vaccine protocols in order to elicit relatively low and high CD4^+^ T cell-mediated IFN-γ responses. We previously reported that vaccination with a plasmid DNA encoding Ag85B—hereafter referred to as DNA vaccination—induced a relative low CD4^+^ T cell-mediated IFN-γ response and a low Ab production in WT C57BL/6 mice [17, 18]. This was associated with protection against Mtb challenge [17]. When DNA primed mice were boosted with adjuvant-free rAg85B protein—hereafter referred to as DNA/protein-vaccination—IFN-γ responses and levels of total IgG and IgG2a/c specific for Ag85B were greatly enhanced [17, 18]. However, this resulted in the loss of protection conferred by DNA-immunization alone [17, 18]. Thus, spleen cells of DNA- and DNA/protein-vaccinated mice, were used in *ex vivo* antigen recall experiments to investigate the contribution of B cells on CD4^+^ T cell cytokine recall responses.

## Materials and Methods

### Ethics Statement

The handling of mice were conducted in accordance with the regulations set forward by the institutional animal care committee of the Italian Ministry of Health, and in compliance with European Community Directive 86/609 and the U.S. Association for Laboratory Animal Care recommendations for the care and use of laboratory animals. The research was approved by the institutional animal care committee of the Italian Ministry of Health (N° 220/2010-B).

### Reagents

Plasmid Ag85B-coding DNA vaccine was prepared by Laura Brunori, Istituto Superiore di Sanità, Rome, as previously reported [17]. The recombinant (r)Ag85B protein (LPS content was below 4.3 EU/μg of protein, by a *Lymulus* amebocyte lysate test) was purchased by Diatheva, (Fano (PU), Italy) and prepared as previously reported [17].

### Immunizations

Wild-type (Charles River Laboratories) and *Igh-6* ^*tm1Cgn*^ C57BL/6 female mice (supplied by Dr. Enrico Proietti, Istituto Superiore di Sanità, Rome, Italy) (6 to 8 week old) were immunized twice at 2-week intervals with 50 μg of plasmid Ag85B-encoding DNA injected intramuscularly in 50 μl PBS into the hind leg (DNA vaccination). Some DNA-primed-mice were boosted twice at 2-week intervals with 10 μg of adjuvant-free rAg85B protein administered subcutaneously on the dorsum of mice (DNA/protein vaccination). As naïve, unvaccinated control group, some mice received only PBS. We used 5 mice per each experimental group. Four separate experiments were performed with WT C57BL/6 mice and 3 separate experiments with *Igh-6* ^*tm1Cgn*^ C57BL/6 mice. Four weeks after the last immunization, mice were sacrificed by cervical dislocation and spleens were harvested for immunological studies.

### Spleen cell preparation and separation of B and CD4^+^ T cells

Single cell suspensions were prepared from pooled spleens recovered 4 weeks after the last immunization. Spleens of mice were aseptically collected and single-cell suspensions created by pressing the spleens through cell strainers. Red blood cells were lysed. Some cells were frozen and stored in liquid nitrogen. A portion of the splenocytes from unvaccinated or vaccinated WT or *Igh-6* ^*tm1Cgn*^ mice were used to isolate CD4^+^ T cells by negative selection magnetic cell separation using a specific mouse CD4^+^ T cell isolation kit (Miltenyi Biotec Inc. Auburn, CA). Similarly, Ag85B-inexperienced B cells were isolated by negative selection magnetic cell separation using a specific mouse B cell isolation kit (Miltenyi Biotec Inc.) from single cell suspensions of spleens of unvaccinated WT mice. The purity of isolated CD4^+^ T cells and B cells ranged between 94% and 96% as estimated by FACS analysis. Unfractionated spleen cells, or co-cultures with purified B and CD4^+^ T cells were used in *ex vivo* antigen recall experiments and analysed for surface and intracellular flwo-cytometry analysis, detection of cytokine production and cell proliferation.

### Cell cultures

Cells were cultured in 96-well plates (3.5x10^5^ cells/well unless stated otherwise for unfractionated spleen cells; 10^5^ cells/well for purified CD4^+^ T cells; 2x10^5^ cells/well unless stated otherwise for purified naive B cells; and 2.5x10^5^ cells/well for naïve spleen cells when used as APC) in RPMI-1640 supplemented with 10% heat-inactivated FBS, 2 mM L-glutamine, 10 mM HEPES buffer, 50 μM 2-β-ME, 50 U/ml penicillin and 50 μg/ml streptomycin. Cells were stimulated with 5 μg/ml rAg85B protein for the indicated periods. In some experiments the antigen was removed from the cultures after 24 hours and rAg85B (5 μg/ml) was re-added or not for further 72 hours. In other experiments, B cells were pre-treated or not with wortmannin (Sigma-Aldrich) (1 μM for 45 min at 37°C) before to be co-cultured with CD4^+^ T cells. In experiments with neutralizing monoclonal (m)Abs, co-culture of CD4^+^ T cells and B cells were stimulated with rAg85B protein (5 μg/ml) in the presence or absence of neutralizing anti-PD-L2 (TY25), anti-PD-L1 (M5/114.15.2), anti-PD-1 (10B1223) or anti-MHC class II (P3U1) mAbs and their matched isotype control mAbs (5 μg/ml each) for 96 hours.

### Cytokine detection in culture supernatants

Supernatants of cultured cells were assayed for cytokine production by specific quantitative sandwich ELISA kits (R&D System, Inc.), in accordance with manufacturer’s instructions.

### Flow Cytometry analysis

Cultured cells were stained using mAb specific for mouse cell surface markers conjugated to FITC, PE, PE-Cy5, PerCP, PE-Cy7 (BD Biosciences Pharmingen; eBioscience, San Diego CA; Serotec LTD, Oxford, UK; BioLegend, San Diego CA; and Immunological Sciences, Rome, Italy) at a concentration of 5 μg/ml in 1%BSA-PBS for 20 min at 4°C. Before staining with specific mAb, the Fc receptors were blocked using anti-FcR (BioLegend) at a concentration of 5 μg/ml for 10 min at 4°C. The following mAbs were used: CD4 (GK1.5; RM4-5) CD8 (53–6.7), CD3 (17A2; 145-2c11), CD69 (H1.2F3), MHC class II (14-4-45), CD279 (PD-1; 29F.1A12), CD274 (PD-L1; 10F.9G2), CD273 (PD-L2; TY25) and B220 (RA3-6B2). Single cell events were gated by forward scatter area versus height and side scatter for size and granularity. Cells were analyzed using a FACSCalibur flow cytometer (Beckton Dickinson Immunocytometry System, San Jose, CA) and Cell Quest analysis software. Staining of samples with isotype controls was used as a reference to determine positive and negative populations.

### Intracellular cytokine staining

For intracellular cytokine staining, cells were treated overnight with 10 μg/ml of brefeldin A (Sigma-Aldrich). Cells were then washed and incubated with Fc block solution (Bio Legend) for 10 min, followed by staining with a PE-Cy5-CD4 mAb for 20 min at 4°C. Cells were then washed and fixed for 5 min with 4% paraformaldehyde. Fixed cells were permeabilized and stained with FITC or PE anti-mouse IFN-γ (XMG1.2) or isotype controls in 1%BSA-PBS buffer with 0.5% saponin for 45 min at room temperature. Cells were then washed in 1% BSA-PBS buffer with 0.1% saponin and subsequently with 1% BSA-PBSbuffer with 0.01% saponin and then analyzed as described in the previous section.

### Cell proliferation by carboxyfluorescein succinimidyl ester staining

Cells (10^7^ cells/ml) were stained with carboxyfluorescein succinimidyl ester (CFSE) (Invitrogen Life Technologies) at 1 μM in PBS 1%FBS for 10 min at 37°C in the dark. Cells were washed and cultured in 96-well plates, as previously described. Four days later, cells were labeled with conjugated mAbs directed against CD3, CD4 and CD8. Cells were then analyzed at flow cytometer as previously described.

### Cell proliferation by [H^3^]thymidine incorporation

Cells were pulsed with [H^3^]thymidine (1μCi/well) (Perkin Elmer Life and Analytical Sciences, Boston, MA) for the last 18 hours of culture. Incorporation of [H^3^]thymidine was measured by β-scintillation counting (Micro β counter, Perkin Elmer), and the incorporation data were expressed as mean count per minute (cpm).

### Statistical analysis

GraphPad Prism 6 Software was used for statistical analysis with Student’s t test, one-way or two-way ANOVA and Tukey’s multiple comparisons post-test. A *p*-value of < 0.05 was considered significant.

## Results

### Ag85B recall activates B cells in spleen cells of vaccinated WT mice

**Fig 1:**
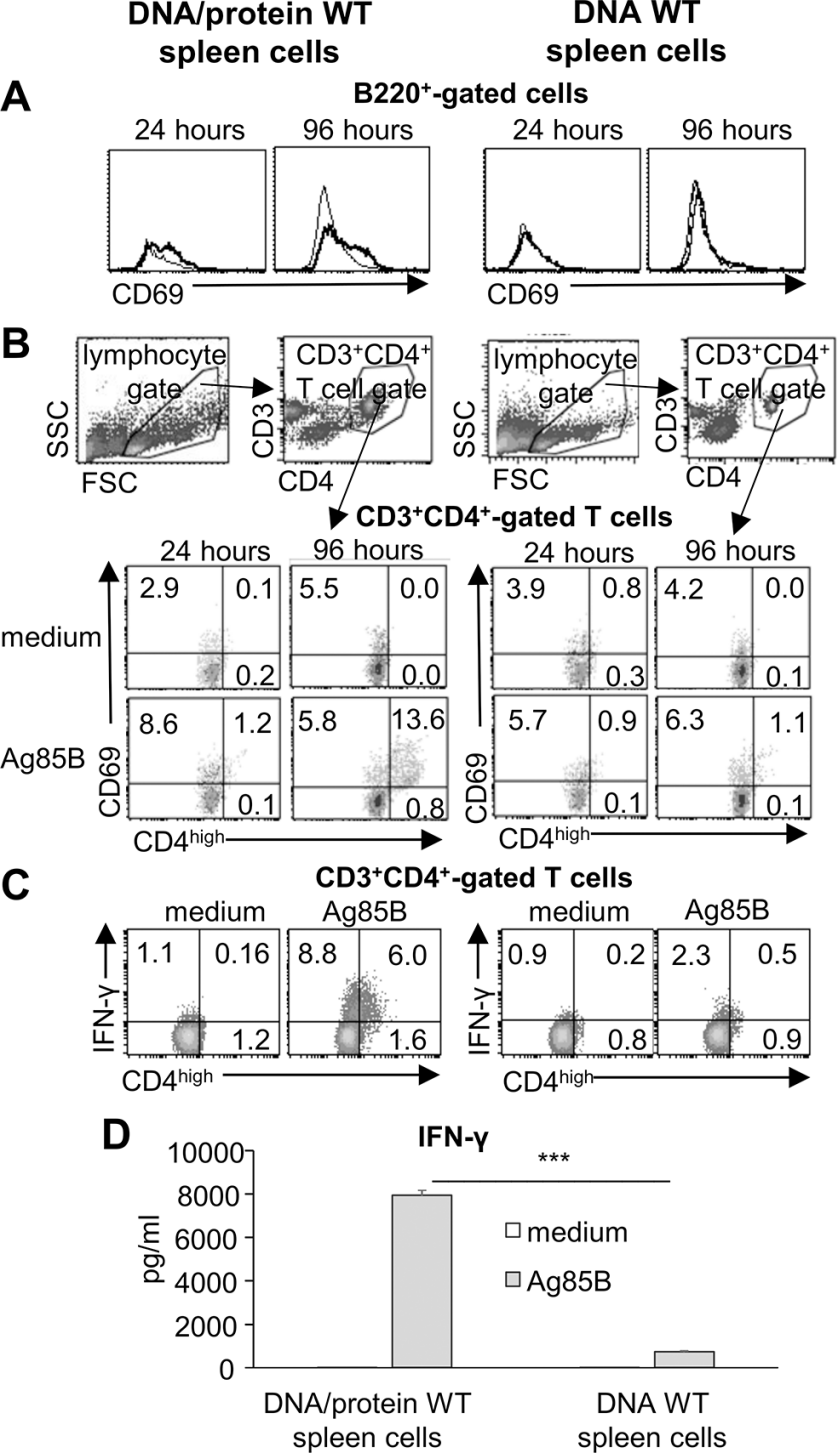
Th1 IFN-γ recall response parallels with B cell activation in spleens of vaccinated WT mice. Unfractionated spleen cells of DNA/protein- or DNA-immunized WT mice were stimulated *ex vivo* with rAg85B protein. (**A**) Flow cytometry analysis for CD69 expression among B cells at 24 and 96 hours; gate on B220^+^ cells, grey histograms: medium culture; bold black histograms Ag85B-stimulated culture. (**B**) Flow cytometry analysis for CD69 expression among CD4^+no high^ and CD4^high^ T cells at 24 and 96 hours; density plot gated on CD3^+^CD4^+^ T lymphocytes. In the top, representative density plots (96 h, medium) showing the gating strategy for identification of CD3^+^CD4^+^ T cells. (**C**) Flow cytometry analysis for intracellular IFN-γ staining on CD4^+no high^ and CD4^high^ T cells at 72 hours; density plot gated on CD3^+^CD4^+^ T lymphocytes. (**A-C**) Data are representative of three independent experiments; in the lefts, plots from DNA/protein WT spleen cells; in the right, plots from DNA WT spleen cells. (**D**) Levels of IFN-γ in culture supernatants at 96 hours. Data are mean of three independent experiments. Error bars, SEM.*,*p*<0.05, **,*p*<0.01, ***, *p*<0.001, ****,*p*<0.0001; two-way ANOVA and Tukey’s post-test.

We asked whether B cells were involved in immune responses to recall Mtb antigens. This was investigated in spleen cells of WT mice vaccinated with DNA and DNA/protein, the two Ag85B-based immunization protocols able to induce relatively low and high IFN-γ responses by CD4^+^ memory T cells, respectively [17, 18]. Spleen cells were recovered after 4 weeks from the last immunization and stimulated *ex vivo* with the rAg85B protein. We observed that in unfractionated spleen cells of DNA/protein-vaccinated mice, B cells expressed the activation marker CD69 after 24 hours of antigen-recall and the CD69 expression was still maintained at 96 hours (Fig 1A).

The B cell activation was associated with the expansion of a highly activated CD4^high^CD69^+^ T cell subset and a relatively high Th1 IFN-γ response (Fig 1B–1D). On the other hand, B cells showed a very modest activation in spleen cells of DNA-vaccinated mice (Fig 1A), in agreement with a relatively low CD4^+^ T cell-mediated IFN-γ response (Fig 1B–1D). These data indicated that the reactivation of Th1 memory cell response in spleen cells of Ag85B-vaccinated mice was associated with the activation of B cells.

### Ag85B-mediated B cell activation is dependent on the magnitude of CD4^+^ memory T cell response

**Fig 2:**
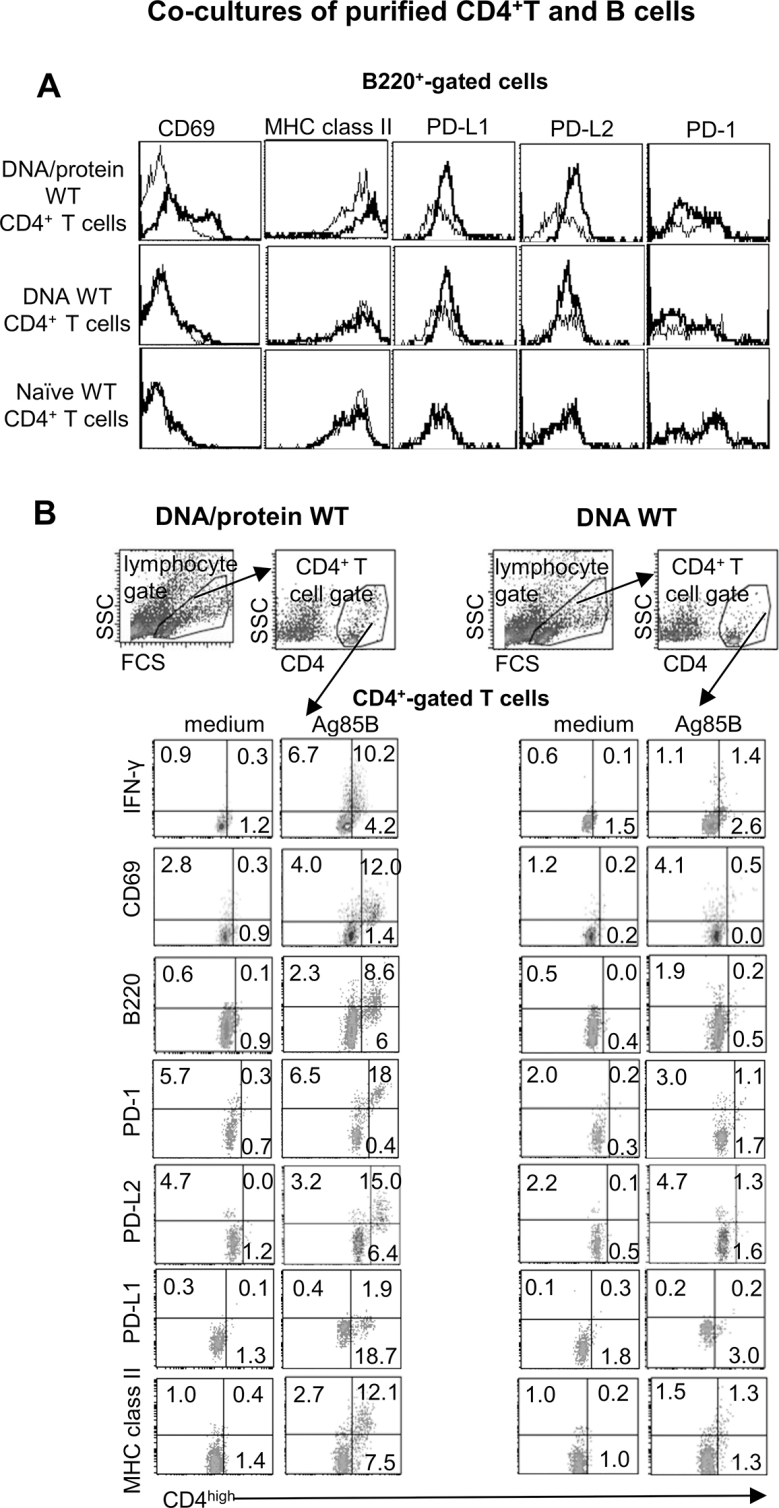
Flow cytometry analysis in Ag85B-stimulated co-cultures of vaccinated WT CD4^+^ T cells and B cells. Inexperienced B cells and CD4^+^ T cells purified from spleens of unvaccinated, DNA/protein- or DNA-vaccinated WT mice were co-cultured and stimulated *ex vivo* with rAg85B protein before flow cytometry analysis. (**A**) Surface expression profiles of the indicated markers on B cells at 96 hours, gate on B220^+^ cells, grey histograms: unstimulated culture; bold black histograms rAg85B-stimulated culture. (**B**) Intracellular IFN-γ staining (at 72 hours) and surface expression (at 96 hours) of the indicated markers on CD4^+no high^ and CD4^high^ T cells, density plots gated on CD4^+^ T lymphocytes. In the top, representative density plots (96 hours, Ag85B stimulation) showing the gating strategy for identification of CD4^+^ T cells. Data are representative of three independent experiments.

To investigate whether B cells were directly involved in the reactivation of memory CD4^+^ T cells we performed co-culture experiments with purified CD4^+^ T cells isolated by spleens of vaccinated and unvaccinated WT mice and Ag85B-inexperienced B cells isolated from spleens of unvaccinated WT mice. The Ag85B-inexperienced B cells—hereafter referred to as inexperienced B cells—have been used to exclude a mandatory requirement of antigen-experienced B cells in interaction with CD4^+^ memory T cells. First, we observed that Ag85B-mediated activation of B cells was triggered by CD4^+^ memory T cells and was associated with the magnitude of T cell response (Fig 2A). In fact, Ag85B protein stimulation was unable to affect B cell phenotype when inexperienced B cells were cultured alone (data not shown) or with purified CD4^+^ T cells isolated from unvaccinated mice (Fig 2A). Instead, CD69, major histocompatibility complex (MHC) class II molecules and both ligands of the Programmed death (PD)-1 receptor, PD-ligand (L)1 and PD-L2 were greatly up-modulated on the surface of inexperienced B cells when cultured with purified DNA/protein WT CD4^+^ T cells (Fig 2A). Inexperienced B cells co-cultured with purified DNA WT CD4^+^ T cells expressed PD-L1, but not PD-L2, and slightly up-modulated CD69, in line with a weaker T memory response (Fig 2A).

### Antigen recall activates CD4^high^MHC classII^+^B220^+^PD-L2^+^ T cells

Second, we found that inexperienced B cells were able to support the reactivation of CD4^+^ memory T cells (Fig 2B). Flow cytometry analysis revealed that antigen recall in DNA/protein WT CD4^+^ T cells co-cultured with inexperienced B cells induced the peculiar CD4^high^ subset high positive for CD69 (Fig 2B), previously observed in Ag85B-stimulated unfractionated DNA/protein WT spleen cells (Fig 1B). Those CD4^high^ T cells, positive to intracellular IFN-γ staining, expressed markers typical of B cells such as B220, MHC class II and PD-L2 (Fig 2B), suggesting that CD4^high^ T cells might have passively acquired them from B cells. It could be true especially for MHC class II molecules since activated murine T cells are unable to express MHC class II for an impairment of CIITA transcription [37]. Intriguingly, those highly activated CD4^high^ T cells were also positive for the exhaustion markers PD-1 (Fig 2B).

In Ag85B-stimulated co-cultures of DNA WT CD4^+^ T cells and inexperienced B cells, the CD4^high^ T cell subset was almost absent and the CD4^+^ T cell population scarcely expressed the above markers, including intracellular IFN-γ, in agreement with their lower activation (Fig 2B).

Thus, B cells, not necessarily Ag85B-experienced, were important actors in the reactivation of Th1 memory cells with the establishment of a positive-feedback loop of mutual activation between B and T lymphocytes.

### The absence of B cells in APC population reduces the IFN-γ recall response by Ag85B-specific CD4^+^ T cells

**Fig 3:**
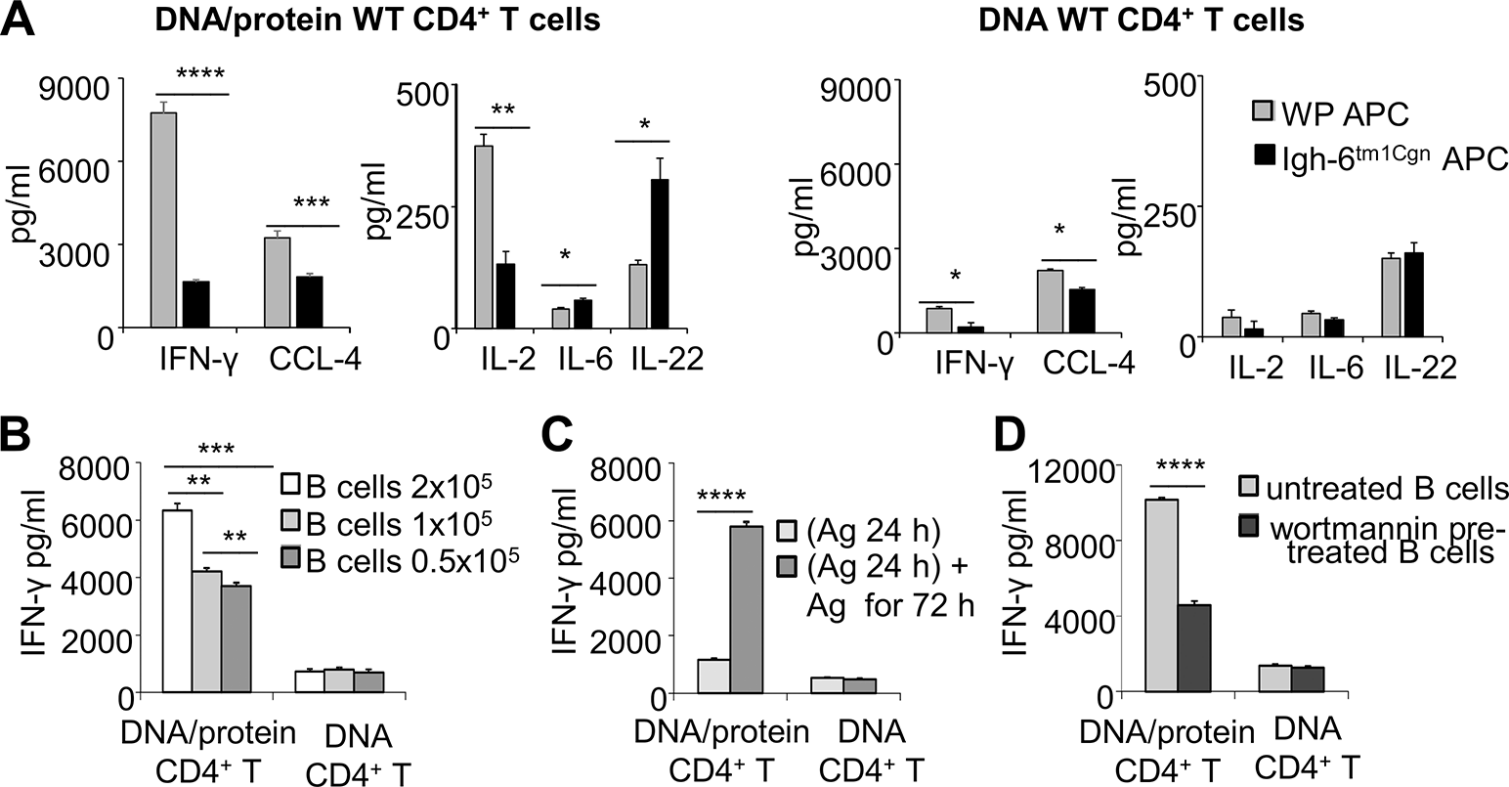
Maximal IFN-γ recall response by Ag85B-specific CD4^+^ memory T cells requires B cells. (**A**) Levels of cytokines in co-cultures of purified CD4^+^ T cells recovered from spleen cells of DNA/protein- or DNA- immunized WT mice and naive WT or *Igh-6* ^*tm1Cgn*^ spleen cells as source of APC. Cells were stimulated *ex vivo* with rAg85B protein for 96 hours, except for IL-2 measured at 24 hours. Naïve WT and *Igh-6* ^*tm1Cgn*^ spleen cells did not release detectable amounts of cytokines upon Ag85B stimulation (data not shown). (**B**) IFN-γ levels in co-cultures of purified CD4^+^ T cells recovered from spleen cells of DNA/protein- or DNA- immunized WT mice and different numbers of inexperienced B cells upon rAg85B protein stimulation for 96 hours. (**C**) IFN-γ levels in co-cultures of purified CD4^+^ T cells recovered from spleen cells of DNA/protein- or DNA- immunized WT mice and inexperienced B cells. Cells were stimulated for 24 hours with rAg85B protein, and after antigen removal, cultured for further 72 hours in absence or in the presence of the antigen. (**D**) IFN-γ levels in co-cultures of purified CD4^+^ T cells recovered from spleen cells of DNA/protein- or DNA- immunized WT mice and inexperienced B cells pre-treated or not with wortmannin. Cells were stimulated with rAg85B protein for 96 hours. Purified CD4^+^ T cells and inexperienced B cells cultured alone did not release detectable amounts of cytokines upon Ag85B stimulation (data not shown). (**A-D**) Data are mean of three independent experiments. Error bars, SEM.*,*p*<0.05, **,*p*<0.01, ***, *p*<0.001, ****,*p*<0.0001; two-way ANOVA and Tukey’s post-test.

We asked the role played by inexperienced B cells in promoting IFN-γ recall response by CD4^+^ memory T cells. First, the effects of B cells were studied in the context of entire spleen cell population by using, as antigen presenting cells (APC), spleen cells of unvaccinated WT and *Igh-6* ^*tm1Cgn*^ mice, the latter lacking mature B cells. Purified DNA/protein WT CD4^+^ T cells produced higher levels of IFN-γ when Ag85B protein was presented by spleen cells of WT compared to *Igh-6* ^*tm1Cgn*^ unvaccinated mice (Fig 3A). Also chemokine (C-C motif) ligand 4 (CCL-4), an inflammatory Th1 associated chemokine, and to a lower extent interleukin (IL)-2 accumulated in greater amount in supernatant when B cells composing the APC population (Fig 3A). Instead, the lack of B cells favored production of IL-22, a member of the IL-10 family and an effector cytokine of the Th17 lineage [38] with a still uncertain role in TB protection [38–42]. IL-6, a cytokine relevant in the IL-17/IL-22 axis, was enhanced, too (Fig 3A). The absence of mature B cells reduced also IFN-γ and CCL-4 production by DNA WT CD4^+^ memory T cells (Fig 3A). No cytokines were produced by both unvaccinated WT and *Igh-6* ^*tm1Cgn*^ spleen cells upon Ag85B stimulation (data not shown), indicating that cytokine production was due to CD4^+^ T cells of vaccinated WT mice.

These data indicated that the type of APC influenced and shaped the quality and magnitude of cytokine recall responses by CD4^+^ memory T cells.

### Maximal IFN-γ recall response is dependent on B cell size, antigen availability and phosphatidylinositol-3 kinase signaling

Then, we investigated the direct influence of inexperienced B cells on CD4^+^ T cell-mediated IFN-γ recall response. To ascertain whether a critical number of B cells was required for maximal IFN-γ production, purified CD4^+^ T cells of immunized WT mice were stimulated with the antigen in the presence of increasing numbers of inexperienced B cells. We observed that IFN-γ production by Ag85B-specific CD4^+^ T cells of DNA-protein-, but not of DNA-immunized WT mice was dependent on B cell number (Fig 3B). Then, antigen-removal experiments were performed to study whether the availability of extracellular antigen can affect the efficiency of B cell-mediated antigen presentation. We found that a continuous availability of extracellular antigen in the culture medium was required for maximal secretion of IFN-γ exclusively by CD4^+^ T cells of DNA/protein-immunized WT mice (Fig 3C). Finally, we investigated whether the integrity of phosphatidylinositol-3 kinase (PI3K) signaling pathways, involved in several active B cell processes related to antigen presentation/co- stimulation [43–46] was also required. We found that pre-treatment of inexperienced B cells with the PI3kinase inhibitor wortmannin significantly inhibited the production of IFN-γ by high-responsive, but not low-responsive, memory CD4^+^ T cells (Fig 3D). These data indicated that the antigen presentation by inexperienced B cells had a primary role in potentiating IFN-γ production by high IFN-γ producer memory CD4^+^ T cells.

### Neutralization of PD-L2 signaling inhibits IFN-γ production and activation of B and CD4^high^ T cells

**Fig 4:**
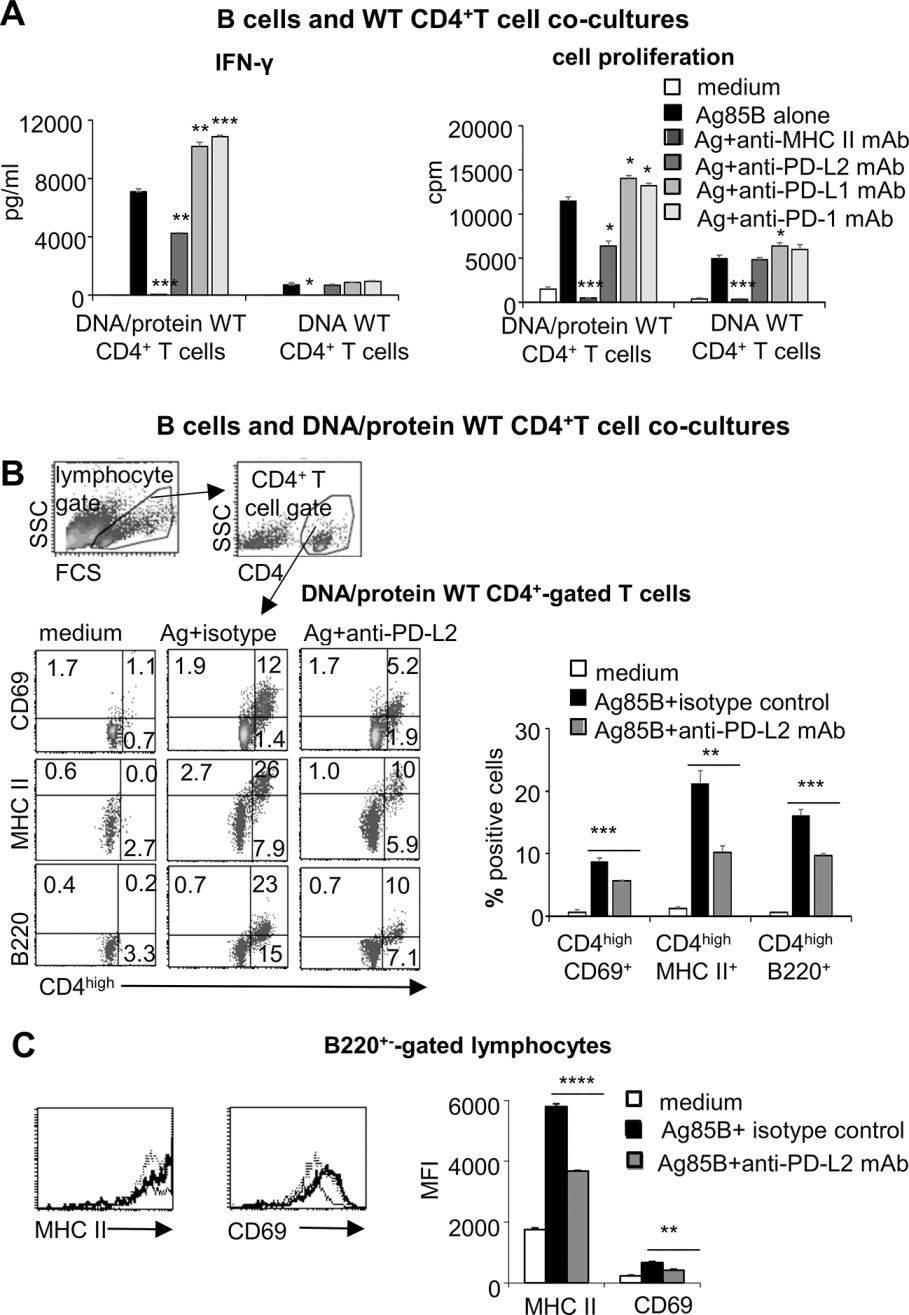
PD-L2 signaling enhances cell activation and IFN-γ recall response by DNA/protein WT CD4^+^ T cells. Inexperienced B cells and CD4^+^ T cells purified from spleens of DNA/protein (**A- C**)- or DNA (**A**)- immunized WT mice were co-cultured and stimulated *ex vivo* with rAg85B protein in the presence of neutralizing anti-PD-L2, anti-PD-L1, anti-PD-1 and anti-MHC class II mAbs or their matched isotype control mAbs for 96 hours. (**A**) IFN-γ levels in culture supernatants and proliferative response as ^3^[H]-Thymidine incorporation. Isotype matched control mAbs did not influence Ag85B-stimulated responses (data not shown). Data are mean of three independent experiments. Error bars, SEM. *,*p*<0.05, **,*p*<0.01, ***,*p*<0.001, ****,P<0.0001, rAg85B stimulation versus antigen stimulation in the presence of neutralizing mAbs; one-way ANOVA and Tukey’s post-test. (**B**) Flow cytometry analysis for expression of CD69, MHC class II and B220 among CD4^+no high^ and CD4^high^ T cells in co-cultures of DNA/protein WT CD4^+^ T cells and inexperienced B cells. Representative density plot gated on CD4^+^ T lymphocytes. In the top, representative density plots (96 hours, medium) showing the gating strategy for identification of CD4^+^ T cells. The graph showing the means of percentages of CD4^high^ cells positive for the indicated markers among CD4^+^ T cells. Data are mean of three independent experiments. Error bars, SEM. **,*p*<0.01, ***,*p*<0.001, one-way ANOVA and Tukey’s post-test. (**C**) Flow cytometry analysis for expression of CD69 and MHC class II on inexperienced B cells co-cultured with DNA/protein WT CD4^+^ T cells and stimulated as described above. Representative histograms gated on B220^+^ cells, grey histograms: unstimulated culture; bold black histograms stimulation with rAg85B and isotype control, dotted histograms: stimulation with rAg85B and anti-PD-L2 mAb; the graph showing the means of CD69 and MHC class II mean fluorescence intensity (MFI) surface expression among B cells. Data are mean of three independent experiments. Error bars, SEM. **,*p*<0.01, ****,P<0.0001; one-way ANOVA and Tukey’s post-test.

We asked whether the reactivation of Ag85B-specific CD4^+^ T cells mediated by inexperienced B cells was MHC class II-restricted. A neutralizing anti-MHC class II mAb completely inhibited IFN-γ production and cell proliferation in all co-cultures of immunized WT CD4^+^ T cells and inexperienced B cells, indicating that antigen presentation via MHC class II was essentially required in recall responses (Fig 4A).

Then, we investigated the contribution of PD-1/PD-Ls molecules in antigen recall responses, taking into account that Ag85B-stimulation affected PD-1/PD-Ls expression on surface of both B and CD4^+^ T cells. Thus, specific neutralizing mAbs and their isotype-matched controls were used in co-cultures of purified immunized WT CD4^+^ T cells and inexperienced B cells. Neutralization of PD-1 receptor increased both cell proliferation and IFN-γ production in co-cultures of DNA/protein WT CD4^+^ T cells (Fig 4A), in accordance with the inhibitory signals triggered by this death receptor to shutdown T cell response [47, 48]. However, blocking signals mediated by the two PD-1 ligands triggered opposite effects. Signaling through PD-L1 was in the same direction as that of PD-1 and its blocking enhanced the T cell responses (Fig 4A). Instead neutralization of PD-L2 inhibited IFN-γ production (Fig 4A), cell proliferation (Fig 4A) and activation of both the CD4^high^ T cells–as indicated by the lower percentage of cells expressing CD69, MHC class II and B220 (Fig 4B)- and B cells which down-modulated MHC class II and CD69 expression (Fig 4C). Thus, PD-L2, differently from PD-L1, did not mediate inhibitory signals but acted as a potent co-stimulus in the mutual activation between B and CD4^+^ memory T cells.

Blocking PD-L1 signaling in Ag85B-stimulated co-culture of DNA WT CD4^+^ T cells determined an enhancement of cell proliferation (Fig 4A) that well fitted with the inhibitory role of the molecule and its expression on B cells (Fig 2A and 2B). On the contrary, a neutralizing anti-PD-L2 mAb, was ineffective in modulating both proliferation and IFN-γ release (Fig 4A), in accordance with the low/absent expression of PD-L2 molecule (Fig 2B and 2C).

### Ag85B-specific IFN-γ production is blunted and IL-22 enhanced in vaccinated *Igh-6* ^*tm1Cgn*^ mice

**Fig 5:**
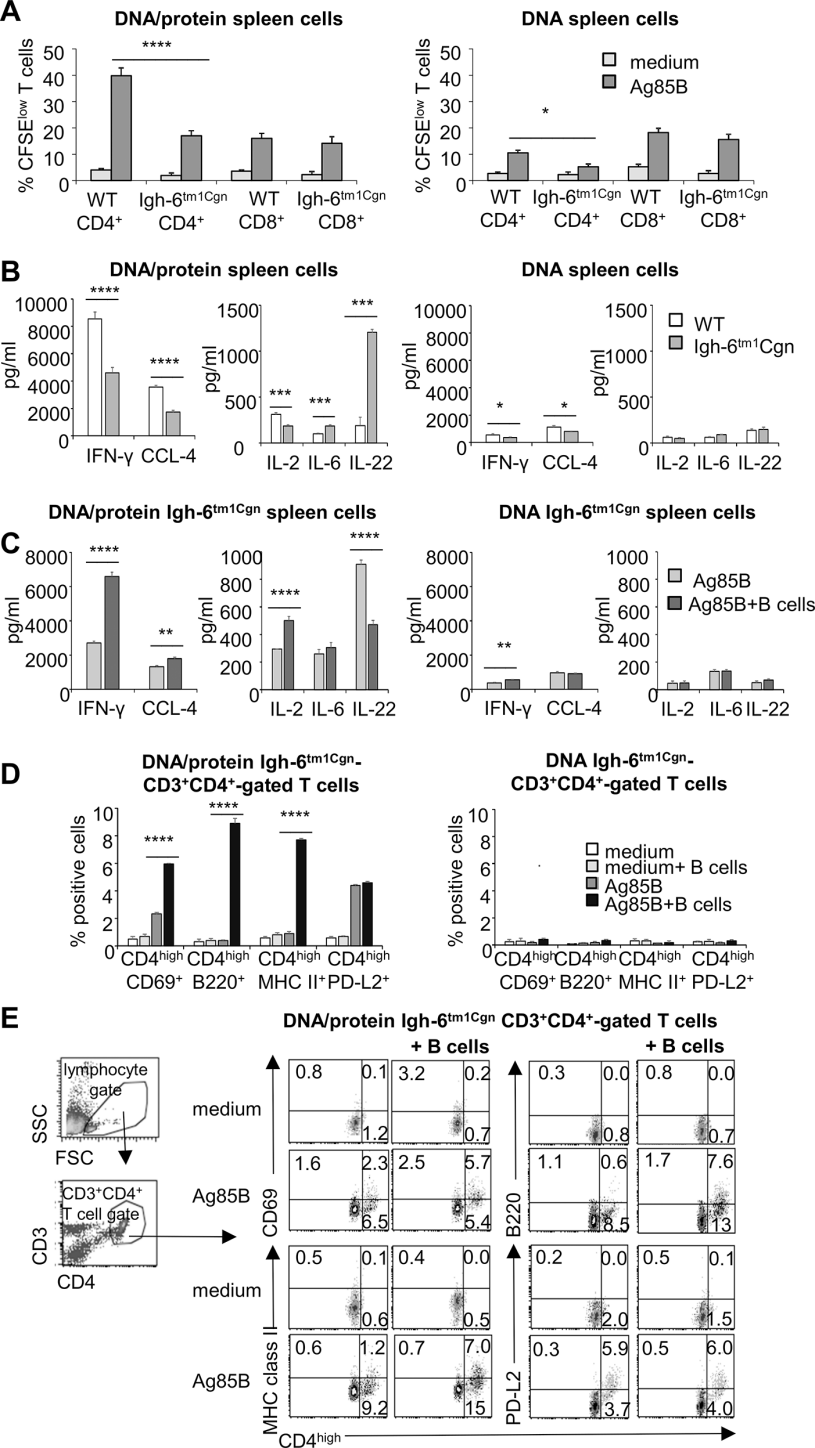
B cells restore IFN-γ recall responses blunted in spleen cells of vaccinated *Igh-6* ^*tm1Cgn*^ mice. (**A** and **B**) Spleen cells of DNA/protein- or DNA-immunized WT or *Igh-6* ^*tm1Cgn*^ mice (4x10^5^ cells/well) were stimulated *ex vivo* with rAg85B protein for 96 hours. (**A**) Percentages of CFSE^low^ cells, as measure of CD4^+^ and CD8^+^ T cell proliferation, and (**B**) levels of cytokines in culture supernatants; IL-2 was measured at 24 hours. No cytokines were detected in unstimulated cultures (data not shown). Data are mean of four independent experiments. Error bars, SEM. *,*p*<0.05, **,*p*<0.01, ***,*p*<0.001; ****,*p*<0.0001 differences between Ag85B-induced responses in spleen cells from vaccinated WT versus *Igh-6* ^*tm1Cgn*^ mice were determined by two-way ANOVA and Tukey’s multiple comparisons test including values of unstimulated cultures not shown in the graphs. (**C-E**) Spleen cells of DNA/protein- or DNA-immunized *Igh-6* ^*tm1Cgn*^ mice (3.5x10^5^ cells/well) were stimulated *ex vivo* with rAg85B protein in the presence or absence of inexperienced B cells (1.5x10^5^ cells/well). (**C**) Levels of cytokines in culture supernatants at 96 hours; except for IL-2 measured at 24 hours. Unstimulated cultures or Ag85B-stimulated B cells cultured alone did not produce detectable amounts of cytokines (data not shown). Data are mean of three independent experiments. Error bars, SEM. *,*p*<0.05, **,*p*<0.01, ***,*p*<0.001; ****,*p*<0.0001, two-way ANOVA and Tukey’s multiple comparisons test including values of unstimulated cultures not shown in the graphs. (**D** and **E**) Flow cytometry analysis for expression of CD69, MHC class II, B220 and PD-L2 on CD3^+^CD4^+^ T cells at 96 hours. (**D**) Percentage of CD4^high^ cells positive for the indicated markers among CD3^+^CD4^+^ T cells in spleen cells of both DNA/protein- and DNA-vaccinated *Igh-6* ^*tm1Cgn*^ mice. Data are mean of three independent experiments. Error bars, SEM. *,*p*<0.05, **,*p*<0.01, ***,*p*<0.001; ****,*p*<0.0001; one-way ANOVA and Tukey’s post-test. (**E**) Representative dot plots for expression of the indicated markers on CD4^+no high^ and CD4^high^ T cells in spleen cells of DNA/protein-immunized *Igh-6* ^*tm1Cgn*^ mice, gate on CD3^+^CD4^+^ T lymphocytes. In the left, representative density plots (96 hours, medium) showing the gating strategy for identification of CD3^+^CD4^+^ T cells.

Next, we examined the impact of B cell compartment on generation of T memory cells by comparing Ag85B-specific responses in WT versus *Igh-6* ^*tm1Cgn*^ vaccinated mice. In spleen cells of DNA/protein-immunized mice stimulated *ex vivo* with rAg85B protein, proliferation of CD4^+^ T cells was significantly reduced in *Igh-6* ^*tm1Cgn*^ compared to WT mice, while CD8^+^ T cells of *Igh-6* ^*tm1Cgn*^ mice proliferated to the same rate as those of the WT mice (Fig 5A). Production of IFN-γ, CCL-4 and IL-2 was blunted, too (Fig 5B). On the other hand, the levels of IL-6 and especially of IL-22, were enhanced in B cell deficient mice (Fig 5B), suggesting a mutual negative influence in antigen recall activation between Th1 and Th22 responses. Ag85B-specific responses elicited by DNA-immunization were similar in *Igh-6* ^*tm1Cgn*^ compared to WT mice, except for a slight but significant reduction in CD4^+^ T cell proliferation and production of IFN-γ and CCL-4 (Fig 5A and 5B). In our Ag85B-based vaccinations, IFN-γ was exclusively produced by CD4^+^ T cells [17, 18, 49], which were also responsible for IL-22 production [50]. Altogether, the data suggested that the absence of mature B cells attenuated the Ag85B-specific Th1 responses especially in DNA/protein vaccination, which, as in WT mice, elicited the higher Ag85B-specific IFN-γ response.

### B cells enhance IFN-γ recall response and activation of Ag85B-specific *Igh-6* ^*tm1Cgn*^ CD4^+^ T cells of DNA/protein-vaccinated *Igh-6* ^*tm1Cgn*^ mice

We asked whether the presence of B cells during antigenic recall could improve the reduced IFN-γ response of vaccinated *Igh-6* ^*tm1Cgn*^ spleen cells. Addition of inexperienced B cells to Ag85B-stimulated DNA/protein *Igh-6* ^*tm1Cgn*^ spleen cells caused a marked increase in production of IFN-γ (about 2.5 fold), IL-2 (about 1.7 fold) and CCL-4 (about 1.35 fold) while decreased IL-22 (about 2.0 fold) (Fig 5C). Overall, the ratio between the amounts of IFN-γ and IL-22 produced in the presence or absence of B cells was around 14 and 3, respectively. Moreover, B cell addition to DNA/protein *Igh-6* ^*tm1Cgn*^ spleen cells determined up-modulation of CD69, in line with an improved activation, and induction of MHC class II and B220 molecules as signs of interactions with B cells, on the CD4^high^ T cell subset (Fig 5D and 5E). On the other hand, expression of PD-L2 on the CD4^high^ T cell subset was not influenced by the addition of B cells, indicating that CD4^+^ T cells did not require B cells to express this ligand of PD-1. Altogether, these data indicated that B cells allowed the *Igh-6* ^*tm1Cgn*^ memory Th1 cells to take action at their best and reduce the gap in IFN-γ production against antigen recall respect to WT CD4^+^ T cells. This suggest that the defective Th1 response in vaccinated *Igh-6* ^*tm1Cgn*^ mice was not attributable to an impairment of CD4^+^ memory T cell compart.

Similarly, to WT mice, spleen cells of DNA-immunized *Igh-6* ^*tm1Cgn*^ mice expressed few CD4^high^ T cells in response to antigen recall. Addition of inexperienced B cells weakly increased the IFN-γ production (Fig 5C) and did not significantly affect CD4^+^ T cell phenotype (Fig 5D).

## Discussion

Here we discover that B cells determine quality and size of T cell cytokine responses against *ex vivo* antigen recall. In line with the emerging ability of B cells to enhance Th1 response during infections [27, 34], we observed that B cells, not necessarily antigen-experienced, maximized IFN-γ recall response by splenic CD4^**+**^ memory T cells specific for Ag85B, a crucial Mtb antigen with a strategic role in host response and TB vaccination [17–21].

In our experimental settings, MHC class II, PI3K signaling and PD-L2 co-stimulation had a contributory role in B cell-mediated amplification of IFN-γ memory response. Continuous MHC contact is necessary for the maintenance of memory CD4^+^ T cell responsiveness to antigen presented by non-professional APCs, such as B cells [51]. Accordingly, maximal IFN-γ production by DNA/protein WT CD4^+^ T cells required continuous extracellular antigen supply and elevated B/CD4^+^ T cell ratios. Of interest, the role of B cells as APC for memory Th1 responses was also shown in mice with targeted deletion of MHC class II in B cells. These mice displayed a reduction of IL-2 and IFN-γ by CD4^+^ memory T cells during *Salmonella* challenge [27] and low pulmonary Th1 cell counts during *Pneumocytis* infection [52]. In addition to canonical expression on APCs, MHC class II molecules were also found on activated Ag85B-specific CD4^high^ T cells, which likely acquired them by capturing B cell membrane portions. Trogocytosis and absorption of exosomes shed from APCs, common in immune cells, allow the transfer of membrane fragments and surface proteins among cells [53, 54]. Thelper cells with captured peptide-MHC class II molecules become effector cells that manifest better recall responses [55]. Some support to this concept comes from the observations that DNA/protein *Igh-6* ^*tm1Cgn*^ CD4^high^ T cells produced higher levels of IFN-γ when expressed MHC class II after interactions with functional B cells. In the same direction are the experiments showing that a reduced expression of MHC class II on DNA/protein WT CD4^high^ T cells paralleled with a reduced IFN-γ production. Moreover, the requirement of a functional PI3K pathway in B cells suggests that the capability to drive CD4^+^ Th1 cell responses may take advantage from those processes related to antigen-presentation/co-stimulatory functions, such as proper phagosomal maturation [45], autophagy [46] but also exosome secretion and assembly of peptide-MHC class II complexes [43, 44].

Binding of death receptors on activated/exhausted T cells is a common self-mechanism to turn off T cell responses. It is no exception the interaction of PD-1, usually found on surface of activated T cells, with its ligands PD-L1 and PD-L2 expressed on APCs [47, 48]. In line with this, we observed that blocking PD-1 and PD-L1 signaling enhanced the activation and IFN-γ production by CD4^**+**^ memory T cells. However, PD-L2 was not involved in down-modulation of T cell functions, but was a potent co-stimulus in the B cell-mediated reactivation of IFN-γ memory response. The unusual abilities of PD-L2 in triggering T cell activation and cytokine production have been previously reported [56]. Moreover, PD-L2 co-stimulation seemed not involve the PD-1 co-receptor. In line with this, mutants of PD-L2 unable to bind PD-1 express positive co-stimulatory functionality [57]. PD-1/PD-Ls axis plays an important role in TB. Both PD-1 and PD-L2 molecules are expressed on T lymphocytes of TB patients and are associated with T cell capability to secrete high levels of IFN-γ especially in high responders versus low responders [48]. In Mtb-infected mice, expression of PD-1 on T cells is associated both with proliferating cells capable to maintain antigen-specific effector T cells during chronic infection [58] and with exhaustion of memory T cell response [59]. Moreover, in humanized mice infected with mycobacteria the majority of CD4^**+**^ T cells express high levels of PD-1 and are defective in mycobacterial control [60].

Peculiar CD4^high^ T cells were involved in high IFN-γ production and interactions with B cells. The increased surface density of the CD4 marker is a trait used to identify antigen-activated and proliferating T cells [61], especially those with a Th1 phenotype [62] and the pathogenic cells found in autoimmune lesions [63]. However, these over-activated effector cells, expressed molecules such as PD-1 [47, 48] and B220 [64] capable of promoting, or associated with, apoptosis and shutting down of the T cell response. Further studies are necessary to understand whether the B cell-mediated massive activation of IFN-γ producing CD4^+^ memory T cells in response to Ag85B recall may ultimately lead to their exhaustion.

B cells mainly affected activation of high, compared to low, IFN-γ producer CD4^**+**^ memory T cells, likely because of the mutual activation and the establishment of a positive loop among B and T cells. Ag85B-stimulated B cells required memory T cells for activation and this paralleled with the magnitude of Th1 response. IFN-γ produced by Ag85B-specific CD4^**+**^ memory T cells is likely to have contributed to B cell activation. Indeed, IFN-γ enhances B cell antigen-presenting/costimulatory functions by up modulating the expression of MHC class II [65] and PD-L2 [66].

While reactivation of IFN-γ recall response was enhanced by B cells, these lymphocytes appeared to negatively influence the release of IL-22. Ag85B-stimulated spleen cells of DNA/protein-vaccinated *Igh-6* ^*tm1Cgn*^ mice released higher levels of IL-22 and showed a lower IFN-γ/IL-22 ratio compared to WT cells. The addition of inexperienced B cells restored the little efficiency of *Igh-6* ^*tm1Cgn*^ spleen cells in promoting the IFN-γ response, but at the same time reduced the IL-22 response. In addition, purified WT DNA/protein CD4^+^ T cells released higher amount of IL-22 when Ag85B protein was presented in the absence of B cells. However, further studies are required to better dissect the role and mechanisms played by B cells in regulating IL-22 recall response. Host immunity to Mtb infection is characterized by the production of both IFN-γ and IL-22, but while the essential role for IFN-γ in resistance to Mtb infection is undoubted [2, 3], the significance of IL-22 in TB remains unclear [38–42]. Of note, in patients with active TB, IL-22 is depleted under conditions of antigen-driven enhanced IFN-γ production by CD4^+^ T cells [67].

The B cell-mediated amplification of IFN-γ production by CD4^**+**^ memory T cells in response to *ex vivo* antigen recall described here, may occur *in vivo* and at the site of infection, too. Therefore, this immune regulatory mechanism may affect both efficacy of TB vaccination and outcome of Mtb infections. For example, besides the B cell regulatory function to inhibit neutrophil migration [34, 35], the absence of mutual activation among B and CD4^**+**^ memory T cells against antigen recall could have contributed to the impairment of Th1 response observed in B cell-deficient mice vaccinated with BCG [34, 35]. It is emerging that B cells are involved in TB immunity more than previously thought. It is not a coincidence that, activated B cells are prominent components of granuloma lesion in the lungs of TB patients and large aggregates of these lymphocytes are relevant histological aspects of the infection [68]. In principle, the influence of B cells on cytokine responses may favor either the host or the pathogen, depending on the peculiar features and circumstances underlying the different phases of Mtb infection. In this regards, B cell-deficient mice develop a more severe pathology, associated with increased IL-17 response and neutrophilia, during the acute phase of infection [34, 35], but the pathogenic inflammatory response is reduced during the chronic one [36].

In conclusion, our data point out the importance of APC nature in determining quality and size of T cell cytokine recall responses. If this is true not only for mice but also for humans, APCs, including B cells, deserve to be considered for a better prediction of cytokine recall responses in blood of TB patients and/or vaccinated subjects. Of note, IFN-γ produced by peripheral CD4^+^ T cells in response to recall Mtb antigens is commonly used for diagnosis of latent/active infections [7, 8], detection of clinical progression [9–12] and evaluation of vaccine immunogenicity [4–6]. Moreover, the host APC compart, including the mechanisms involved in the mutual B-T cell activation, such as PD-L2 and PI3K signaling, could be novel targets for modulation of T memory cytokine responses. All this is relevant for the search of successful vaccines and immunotherapeutic treatments to control TB.

## Supporting Information

S1 ARRIVE Checklist The ARRIVE guidelines checklist. (PDF)

## Abbreviations:

APC: Antigen presenting cells
BCG: bacille Calmette-Guérin
CFSE: carboxyfluorescein succinimidyl ester
CCL-4: chemokine (C-C motif) ligand 4
cpm: count per minute
IL-: interleukin-
IFN-γ: Interferon-γ
MHC: major histocompatibility complex
mAb: monoclonal antibody
Mtb: Mycobacterium tuberculosis
PI3K: phosphatidylinositol-3 kinase
PD-L: Programed death-ligand
r: recombinant
Th: Thelper
TB: tuberculosis
WT: wild-type
